# Risk of pneumothorax in Birt-Hogg-Dubé syndrome during pregnancy and birth

**DOI:** 10.3389/fmed.2023.1289948

**Published:** 2023-11-08

**Authors:** Ortrud K. Steinlein, Marlene Reithmair, Zulfiya Syunyaeva, Elke C. Sattler

**Affiliations:** ^1^Institute of Human Genetics, University Hospital, LMU Munich, Munich, Germany; ^2^Department of Medicine V, University Hospital, LMU Munich, University of Munich, Munich, Germany; ^3^Department of Pediatric Pulmonology, Immunology and Critical Care Medicine, Cystic Fibrosis Center, Charite -Universitätsmedizin Berlin, Berlin, Germany; ^4^Department of Dermatology and Allergy, University Hospital, LMU Munich, Munich, Germany

**Keywords:** Birt-Hogg-Dubè syndrome, pneumothorax, pregnancy, FLCN, birth

## Abstract

Birt-Hogg-Dubé syndrome (BHDS) is a genetic disorder characterized by fibrofolliculomas, renal cell cancer and lung cysts. Patients are at risk to develop pneumothorax but the magnitude of this risk during pregnancy is unknown. Information was obtained from 46 women with BHDS that had at least one pregnancy (BHDS-with preg), 18 female BHDS relatives without pregnancies (BHDS-no preg) and 25 non-BHDS female relatives with at least one pregnancy (noBHDS-with preg). In total, 77 pneumothoraces occurred in the BHDS-with preg group (mean 1.7/patient) and 11 in the BHDS-no preg group. Comparison of patient years for the first two groups showed pneumothorax incidence rates of 0.054 and 0.016, respectively. The incidence rate difference was significant [0.038 (CI 0.02–0.057), value of *p*-value 0.0001]. This difference is not caused by an increased number of patients with pneumothorax but by an increased number of pneumothoraces per patient. Pregnancy in BHDS therefore might be a risk factor for multiple pneumothoraces.

## 1. Introduction

Birt-Hogg-Dubé syndrome (BHDS, MIM: 135150) is a rare genetic tumor syndrome caused by, mostly truncating, mutations in the *FLCN* gene (1, 2). Patients are at risk to develop benign or malignant kidney tumors of different histology (3). Between age 20 and 40 years, most of them start to develop folliculomas, small whitish papules with a mostly smooth surface mainly affecting face and neck. These telltale benign skin tumors tend to increase in number and size over time. Often the earliest symptom in BHDS are multiple lung cysts which are thin-walled, of variable size and morphology and need to be differentiated from other cystic lung diseases such as lymphangiomyomatosis (LAM). At least in some patients, lung cysts are already present in adolescence (if not earlier) but it is not well known at which age they usually start to occur or what the natural course of cysts development is. Recent findings suggest that cysts are growing, contracting, fusing and increasing during lifetime (4). The cysts are mainly located subpleural and in the interlobar pleura at the perilobular area of the lung and it has been demonstrated that they have little communication with airways. It has also been suggested that the lack of communication with airways increases extension in low-pressure conditions, rendering the lung cysts susceptible to rupture during air travel. The risk of pneumothorax during a flight is indeed higher for BHDS than for LAM patients, the latter having predominantly lung cysts that connect to airways (5, 6). The pathophysiology that triggers the development of lung cysts is not well understood and different hypotheses have been presented. It has been proposed that the homeostasis of the alveolar walls might be disturbed and would therefore not be strong enough to resist the mechanical forces caused by breathing. Another hypothesis was presented in the “stretch theory” that is based on the observation that cell–cell adhesion is increased in *FLCN*-deficient cells. This would reduce the flexibility of lung tissue and interfere with its ability to stretch, leading to stretch-induced lung injury (7, 8). Both hypotheses would be able to explain why cysts in BHDS are mainly localized in basal parts of the lung, the area where mechanical forces are most markedly in this organ.

About 44–75% of BHDS patients are affected by spontaneous pneumothorax, caused by ruptured lung cysts (9). While some patients experience their first pneumothorax already in adolescence, most of these events happen between age 20 and 50 years. Thus, the period in life in which pneumothorax risk is at its peak overlaps with the childbearing age of female BHDS patients. This is especially true for patients that suffer from recurrent pneumothorax. In this subgroup the first pneumothorax on average occurs about 10 years earlier (mean 29.2 y), compared to BHDS patients that experience only a single pneumothorax (9, 10). Pneumothorax in pregnancy is therefore an understandable concern for female BHDS patients planning a pregnancy or those already pregnant. However, the frequency of spontaneous pneumothorax in pregnancy has so far not been studied systematically in BHDS patients and therefore no reliable risk estimates exist. In the present study, we addressed this question in a large sample of 101 mostly multiplex BHDS families.

## 2. Materials and methods

### 2.1. Patients and controls

Clinical and pedigree information was available from 108 patients attending the Munich BHDS outpatient clinic between 2005 and 2022 and their relatives. For the present study the patients were again contacted by email or letter, informed about the study and asked to answer a written questionnaire about pregnancies and pneumothorax in the family. Those that answered (53 individuals) were personally contacted by phone or email and asked for details. Included in the study were female patients with at least one pregnancy, female family members without BHDS that had given birth and female family members that were carriers of the *FLCN* mutation but had not given birth. The patients and controls belonged to families of German descent or Eastern European origin with German roots (Volga Germans) and one family of English origin. The study has been approved by the ethical committee/institutional review board (IRB) of the Medical Faculty, University Hospital Munich, under the project-number 508/16UE. All methods were carried out in accordance to the Declaration of Helsinki. The patients consented to participate in the study.

### 2.2. Genetic testing

Informed consent for DNA testing was obtained. Mutation screening of the *FLCN* gene including adjacent intronic sequences was performed as previously described (3, 11).

### 2.3. Statistical analyses

Statistical analyses were performed using the two-tailed Mann–Whitney U test (significance level *p* = 0.05) and the Chi-Square test (significance level *p* = 0.05), with Yates correction for small sample sizes. For the calculation of differences regarding pneumothorax frequency between groups the patient years between age 16–45 years (main child bearing years) were used. Calculation was carried out using the comparison of rates method (12).

Results

Finally, included in the study were 46 female BHDS patients (named *BHDS-with preg*) (present mean age 60.6 years, SD 15.4, range 34–87 years) that had at least one pregnancy (total number of pregnancies: 92). The first control sample (named *BHDS-no preg*) contained female family members that were carriers of the *FLCN* mutation but had not given birth (*n* = 18, present mean age 48.0 years (SD 15.49, range 21–80 years)). For the second control group (named *noBHDS-with preg*) data were collected from 25 female family members (mainly sisters and first-grade cousins) that had given birth (total number of pregnancies: 44) but were not affected by BHDS (*FLCN* mutation excluded either by testing of the control individual or the relevant parent) (present mean age 49.2 years (SD 12.00, range 21–77 years)). This control sample was only used to demonstrate that pneumothoraces are a rare event in the general population; the sample was not used for statistical purposes.

The mean age in the *BHDS-with preg* group was significantly higher than in *BHDS-no preg* group (z-score 2.33, value of p 0.02). However, in both groups the child bearing age was well covered by the mean age.

**Figure 1:**
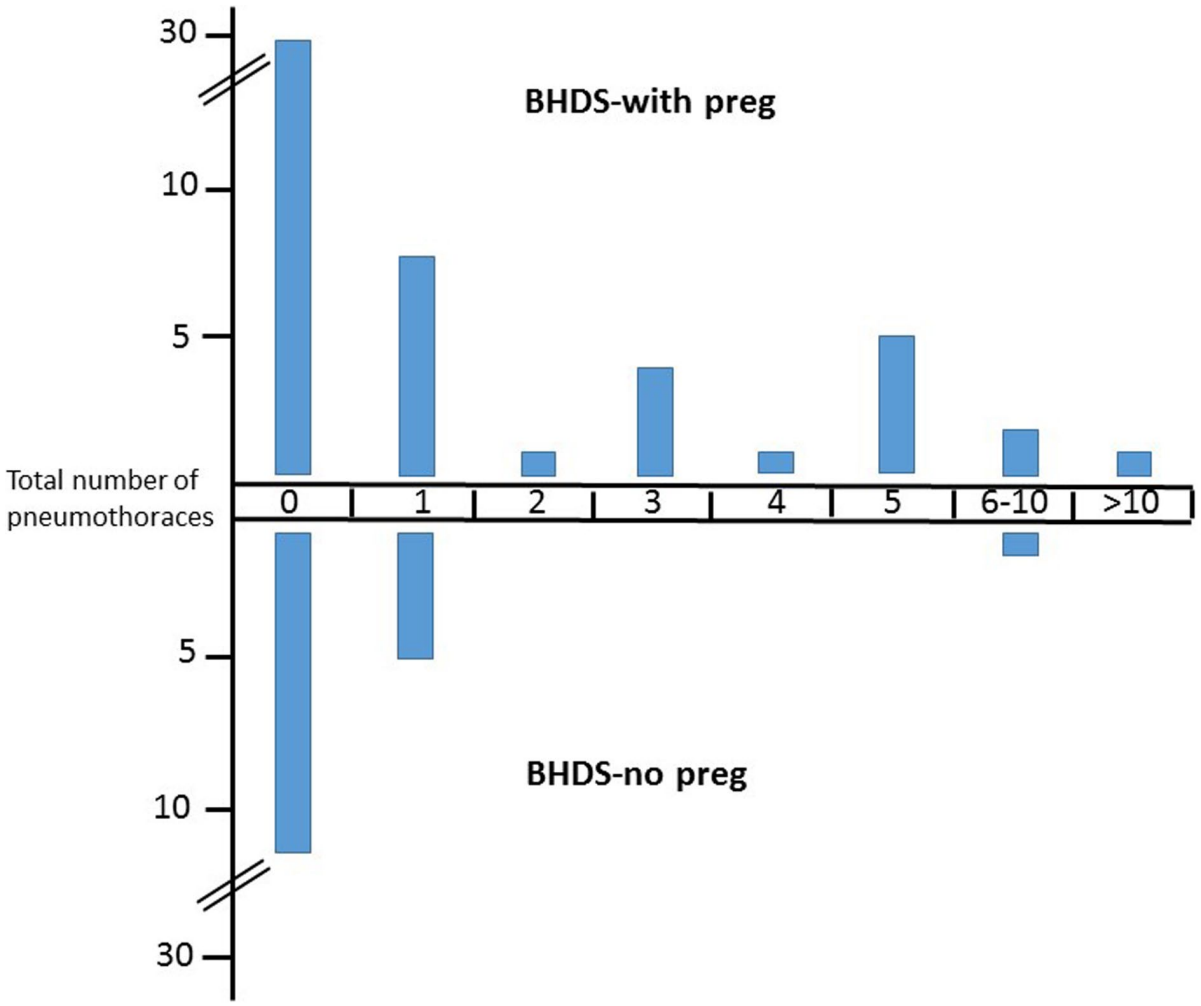
Number of pneumothoraces in BHDS patients with and without pregnancies. Upper part, BHD patient with pregnancies, lower part, BHD patients without pregnancies. *Y*-axis, number of patients.

The pregnancies in the *BHDS-with preg* group occurred at a mean age of 30.2 years (SD 7.10, range 16–49 years) and at a mean age of 30.4 years (SD 4.54, range 20–39 years) in control group *noBHDS-with preg* (*z*-score 0.44, value of *p* 0.66) (Figure 1). The age at first pregnancy was 29.0 years (SD 8.03, range 16–49 years) in *BHDS-with preg* and 29.0 years (SD 4.24, range 20–35 years) in *noBHDS-with preg* (*z*-score − 0.71, value of *p* 0.48). In the *BHDS-with preg* group eight pneumothoraces occurred during pregnancy (mean age 28.3 years, SD 3.67, range 24–36 years). There was no difference with respect to the order of pregnancies; pneumothorax occurred as often in first as in subsequent pregnancies. One of the pneumothoraces during pregnancy occurred in the second month, three in the third one of these was during a twin pregnancy and one happened after a fall down the stairs that resulted in hematomas at the back and lower back (thoracic injuries, especially rip fractures, were not present but mild lung contusion cannot be excluded) and two each in the fourth and seventh month.

For calculation only the main childbearing years between ages 16–45 years were taken into account (*BHDS-with preg* group, 1,417 patient years; *BHDS-no preg,* 691 patient years; *noBHDS-with preg,* 455 patient years). In total, 77 pneumothoraces occurred in the *BHDS-with preg* group (mean 1.7/patient), 11 in the *BHDS-no preg* group and none in the *noBHDS-with preg group*. Comparison of patient years for the first two groups showed incidence rates of 0.054 and 0.016, respectively. The incidence rate difference was significant (0.038 (CI 0.02–0.057), value of p 0.0001).

19 individuals in the *BHDS-with preg* group and 5 patients in the *BHDS-no preg* group suffered at least one pneumothorax. The difference, compared to patient years, was not significant (incidence rates difference 0.006, CI 0.004–0.016, value of p 0.21). Thus the significant difference regarding the total pneumothorax number is obviously not caused by an increased number of patients with pneumothorax in the *BHDS-with preg* group, but by an increased number of pneumothoraces per patient. Indeed, within the *BHDS-with preg* group 12 patients had multiple pneumothoraces, compared to only one patient in the *BHDS-no preg* group (chi square 4.95, value of *p* 0.026).

### 2.4. Medical history of patients with 5 or more pneumothoraces

#### 2.4.1. *BHDS-with preg* group

Patient BHD33-II2: died suddenly for unknown reasons at age 70 years, 6 pneumothoraces in total between age 25 and 65 years, three children, no clinical details about treatment available. Three family members also had multiple pneumothoraces, including her mother (not included in the present study).

Patient BHD39-I2: age 85 years, 5 pneumothoraces in total, 5 pregnancies. No clinical details about treatment available.

Patient BHD42-III2: age 50 years, 8 pneumothoraces in total, first pneumothorax at age 20 years. The second one occurred 4–5 years later and was treated with partial pleurodesis. This procedure was repeated for each but the last of the following pneumothoraces. Cecarian section was used in each of her three pregnancies, no pneumothoraces occurred during pregnancy.

Patient BHD60-II3: age 47 years, 20 pneumothoraces in total, first pneumothorax at age 21 years, the second when she was 4 months pregnant with twins. The pregnancy resulted from *in vitro* fertilization. After this pregnancy she had pneumothoraces every 2–4 years. At age 45 years she again tried *in vitro* fertilization and had several pneumothoraces within the same year. After the 6th pneumothorax she repeatedly had partial pleurodesis.

Patient BHD75-II3: age 69 years, 5 pneumothoraces between age 20–62 years (no clinical details about treatment available), 3 pregnancies. One pneumothorax occurred in early pregnancy after an accidental fall. She has been suffering from severe emphysema for several years now. One of her daughters (4 pneumothoraces total) had pneumothorax when she was 7.-8. months pregnant and a second one two months after giving birth, the second daughter (no children) had 1 pneumothorax at age 42 years and the third daughter (no children) one pneumothorax at age 15 years.

#### 2.4.2. *BHDS-no preg* group

BHD46-III1: age 65 years, 7 pneumothoraces in total, first pneumothorax at age 16 years, last one at age 64 years. Bullae resection and partial pleurodesis was performed for the second and partial pleurodesis for the subsequent pneumothoraces. She developed renal cell cancer bilateral at age 45 years and unilateral at age 57 years.

## 3. Discussion

Spontaneous pneumothorax might occur in patients without any known cause (primary spontaneous pneumothorax) or in those affected by an acquired or inborn lung disorder (secondary spontaneous pneumothorax) (13–15). In clinical practice this distinction is not always clear; structural lung changes such as cysts can also be present in otherwise healthy people. It has been estimated that about 3.4–10% of all spontaneous pneumothoraces occur in patients with BHDS (16). This rare genetic disorder is therefore responsible for a substantial proportion of patients treated for this complication. The lack of reliable clinical markers makes it nevertheless difficult to advise BHDS patients about their individual pneumothorax risk. This is especially a challenge in female BHDS patients of childbearing age; they understandably often worry that a pneumothorax during pregnancy or around birth might put themselves and their unborn child at risk.

The data presented here indicate that pregnancy does not in general increase the risk for pneumothoraces in BHDS patients. However, the number of patients with multiple pneumothoraces was considerably higher in BHDS patients with pregnancies compared to those without. Six of them had five or more pneumothoraces and one even about 20, while in the group of BHDS patients without pregnancies only one woman suffered from multiple events. Thus, it is tempting to speculate that pregnancies, while not constituting a pneumothorax risk factor *per se*, might trigger the occurrence of multiple pneumothoraces in a subgroup of BHDS women that, for unknown reasons, are more at risk. However, independent BHD samples with larger numbers of pregnancies and matching control groups are needed to test this hypothesis. If confirmed, it will be interesting to identify marker, genetic or otherwise, that allow the identification of women for which pregnancy might turn into a trigger for multiple pneumothoraces.

Interestingly, six of the eight pneumothoraces that occurred in pregnant BHDS patients did so in first or second trimester, thus at a time span when the unborn child’s increasing size is not yet likely to interfere with lung extension. Furthermore, none of the pneumothoraces happened during or after childbirth. It is therefore unlikely that something as obvious as mechanical causes or increase of intraabdominal and intrathoracal pressure during birth are important factors influencing the pneumothorax risk in pregnant BHDS patients. A possible explanation for the increased pneumothorax risk in early to mid pregnancy would be changes in hormone equilibrium that are typical for the first trimester. Interestingly, the pneumothorax risk in female BHDS patients in general starts around puberty and decreases markedly around age 50 years (9), a period that is characterized by the hormonal changes of menopause. It is therefore tempting to speculate that lung cysts are destabilized by hormonal factors. Such a mechanism is also supported by the observation that female BHDS patients, compared to males, have a significantly higher residual lung volume as well as significantly lower forced expiratory volume (17). It has therefore been hypothesized that BHDS females in general might have a more severe lung involvement due to hormonal factors. Parallels have been discussed to the cyst formation in LAM that might result from interactions between the estrogen signaling pathway and the mechanistic target of rapamycin (mTOR), in which both the tuberous sclerosis complex and *FLCN* are involved (18, 19).

Four of the eight women that experienced pneumothorax in pregnancy reported that correct diagnosis was considerably delayed. Symptoms caused by the pneumothorax were often at first misinterpreted as muscle pain, pinched nerves or other common minor ailments. Reluctance to employ radiological tests in pregnant women would be an understandable reason for the delay. It is therefore important that medical professionals caring for pregnant BHDS women are aware of the pneumothorax risk and that the presence of symptoms such as shortness of breath, atypical cough or chest pain should promptly initiate clinical evaluation.

In summary, our data indicate that pneumothorax is a rare event in pregnant BHDS women but that pregnancy might put them at an increased risk to suffer multiple pneumothoraces during life time. Furthermore, birth itself does not seem to be a critical phase for pneumothorax since these events mostly occured in first and second trimester. There are some limitations to this study. The retrospective design might have caused overreporting of pneumothorax in pregnancies of data because patients that experienced such a complication might have been more prone to answer the questionnaire. This potential source of bias could at least in part be controlled because we were able to cross-check the returned questionnaire with the extensive data collected when patients visited the outpatient clinic and received genetic counseling. Furthermore, the sample sizes differed and the sample of BHDS patients with pregnancies was twice the size of the control group without pregnancies. Also, miscarriages, which would influence the total number of pregnancies included in the calculation, might not have been reported accurately. Some patients from the *BHDS-no preg* were still young and might have become pregnant later in live. Independent samples of BHDS patients are therefore needed to confirm the observations reported in the present study.

## Data availability statement

The original contributions presented in the study are included in the article/supplementary material, further inquiries can be directed to the corresponding author.

## Ethics statement

The studies involving humans were approved by the LMU University Hospital Ethics committee. The studies were conducted in accordance with the local legislation and institutional requirements. Written informed consent for participation was not required from the participants or the participants’ legal guardians/next of kin in accordance with the national legislation and institutional requirements.

## Author contributions

OS: Data curation, Formal analysis, Writing – original draft. MR: Data curation, Writing – review & editing. ZS: Data curation, Writing – review & editing. ES: Data curation, Writing – review & editing.
